# How Perceived Parental Technoference Relates to Adolescent Smartphone Addiction: Evidence from a Moderated Mediation Model

**DOI:** 10.3390/bs16030363

**Published:** 2026-03-04

**Authors:** Lifen Zhao, Miaomiao Xiao, Binbin Tang, Ya Gao, Dandan Hu

**Affiliations:** 1 Department of Sociology and Social Security, Ginling College, Nanjing Normal University, Nanjing 210097, China; lfzhao@link.cuhk.edu.hk (L.Z.); 241702001@njnu.edu.cn (M.X.); 241702005@njnu.edu.cn (Y.G.); 16220116@njnu.edu.cn (D.H.); 2 Faculty of Philosophy and History (Biquan Academy), Xiangtan University, Xiangtan 411105, China

**Keywords:** perceived parental technoference, attentional shifting, parent–child conflict, smartphone addiction, mindfulness

## Abstract

Previous research has demonstrated a link between perceived parental technoference—defined as parents’ technology-related interruptions during interactions—and adolescents’ smartphone addiction. However, the underlying mechanisms of this relationship remain insufficiently understood. The present study investigates the association between perceived parental technoference and smartphone addiction among Chinese adolescents, with a particular focus on the mediating effects of attentional shifting and parent–child conflict, as well as the moderating role of mindfulness. Data were collected through a questionnaire survey administered to 1084 adolescents in Hebei Province, China. The findings revealed that perceived parental technoference was negatively associated with adolescents’ smartphone addiction, and this relationship was mediated by attentional shifting and parent–child conflict. In addition, mindfulness moderated the relationship between perceived parental technoference and parent–child conflict. These findings help us to gain a deeper understanding of the mechanism in associations between perceived parental technoference and smartphone addiction. They also offer useful implications for social workers and practitioners to develop interventions that effectively reduce smartphone addiction among adolescents.

## 1. Introduction

Smartphones are now an integral part of our lives. According to a survey by the Pew Research Center (2019), over 80% of Americans own a smartphone. In China, the internet is predominantly accessed via mobile devices, with around 1.1 billion users, of whom almost all (99.7%) access the internet via their mobile phones. This is particularly evident among younger people, who are becoming increasingly dependent on their smartphones (Q. Liu et al., 2017). This dependence often manifests as smartphone addiction, which is widely recognized as a behavioral addiction. Its core characteristics include an individual’s loss of control over smartphone use, significant psychological dependence, and subsequent impairments in cognitive and functional domains (Moqbel et al., 2023). Such addiction is associated with many negative outcomes, including sleep problems (Demirci et al., 2015), problems in their social relationships (Seo et al., 2016), and deteriorating mental health (J. Yang et al., 2020).

Research suggests that parental technoference may be a significant factor associated with this adolescent obsession (Shao et al., 2024). Originally introduced by McDaniel (2019), parental technoference refers to the phenomenon in which parents’ use of digital devices interrupts, disturbs, or diminishes the quality of parent–child interactions (Mackay et al., 2022). Within this context, perceived parental technoference specifically describes the adolescent’s subjective perception of these technological interruptions. Social learning theory, as laid out by Bandura (1971), suggests that kids and teenagers acquire behaviors through imitation. When parents are habitually engaged with digital devices, there is a concomitant tendency among their offspring to emulate this behavior, thus raising the likelihood of overusing smartphones. In alignment with this theory, an empirical study (Q. Liu et al., 2020) has demonstrated that perceived parental technoference plays an important role in the development of smartphone addiction among adolescents.

Existing research has identified a significant correlation between perceived parental technoference and teenagers’ overusing smartphones. Nonetheless, the potential pathways that may explain this relationship remain relatively underexplored and warrant further investigation. Drawing on the interactional theory of childhood problematic media use (Domoff et al., 2020), one’s development is influenced by the dynamic interaction between personal attributes and environmental factors. However, empirical support for this theoretical framework in the smartphone addiction field is still limited. Existing studies examining the underlying mechanisms of smartphone addiction tend to focus either on individual factors (Zhang et al., 2021) or family-level factors, such as family relationship (Niu et al., 2020), with few attempts to integrate both factors into a comprehensive framework. Moreover, among studies that have investigated individual mechanisms, the majority have focused on anxiety, sensitivity, or self-control (Q. Liu et al., 2020; Zhang et al., 2021), while comparatively limited attention has been directed toward cognitive variables such as attentional control or attentional shifting, despite evidence linking these factors to smartphone addiction (Domoff et al., 2020; Ge et al., 2023). The present study attempts to addresses these gaps by analyzing how perceived parental technoference relates to adolescent smartphone addiction, with particular attention to the potential mediating roles of attentional shifting and parent–child conflict, while also considering the role of mindfulness as a potential moderator.

### 1.1. Attentional Shifting as a Mediator

Attentional shifting, a subcomponent of attentional control, describes an individual’s capacity to volitionally shift their attention between different tasks (Miyake et al., 2000). According to Domoff et al.’s (2020) theory on problematic media use, smartphone addiction results from the joint influence of environmental factors (e.g., perceived parental technoference) and factors related to individual characteristics (e.g., attentional shifting). When adolescents are exposed to perceived parental technoference, individual cognitive factors (e.g., attentional shifting) could interplay with the technoference, thereby contributing to the adolescents’ smartphone addiction. This suggests that attentional shifting could serve as an intermediary variable between technoference and excessive smartphone use. While Domoff et al.’s (2020) framework hints at this mechanism, there are limited empirical studies. To date, however, only a limited amount of research has examined the mediating role of attentional control. A notable example is provided by Qiao and Liu’s (2020) study, which suggests that attentional control may account for part of the link between perceived parental technoference and problematic smartphone use among adolescents.

Emerging evidence has highlighted a potential link between parental distraction with technology and disruptions in children’s attentional processes. Although most recent research does not provide direct evidence, these studies support the notion that parents’ constant tech use within the family environment is linked to lower levels of adolescents’ attentional shifting. Mobile device usage by parents during interactions with their children tends to create interruptions that may reduce parental responsiveness and contingent feedback, which are critical for scaffolding children’s emerging attentional control capacities. These interruptions may also be related to less effective modeling of sustained focus and self-regulation skills (Kildare & Middlemiss, 2017). A longitudinal study demonstrated that perceived parental technoference significantly predicted poorer attentional control in children, indexed by higher intra-individual reaction time variability during cognitive tasks, reflecting unstable attention regulation (Jiang et al., 2024). This aligns with McDaniel and Radesky’s (2018a) foundational work, which linked perceived parental technoference to child hyperactivity, which is a manifestation of attention problems.

Meanwhile, previous studies have identified an association between attention deficits (e.g., lower levels of attentional shifting) and various forms of digital media overuse (e.g., smartphone addiction). Yoo et al. (2004) found that individuals with attention-related problems (e.g., attention-deficit) tended to report higher levels of internet addiction, indicating that impaired attentional control may contribute to smartphone addiction behaviors. Similarly, B. Q. Wang et al. (2017) discovered that attention deficits and excessive internet use are frequently co-occurring, noting that poor inhibitory control may be linked to greater susceptibility to technology-related addictive behaviors. Supporting this view, Yılbaş and Karadeniz (2022) reported that adolescents exhibiting impulsivity and attention problems were more susceptible to problematic smartphone use, highlighting the important function of attentional regulation in digital media use. People with impaired attentional control may struggle to manage distractions from digital communication media such as smartphones (Hadlington, 2015), which increases their risk of becoming addicted to their smartphones.

### 1.2. Parent–Child Conflict as a Mediator

Perceived parental technoference has been found to disrupt family interactions and give rise to conflicts within the family (Ajilore et al., 2023). Drawing from a well-established conceptualization, parent–child conflict is characterized by behavioral opposition or overt disagreement between a parent and a child (Laursen et al., 1998). When parents are regularly absorbed in digital devices during interactions, adolescents report experiencing more strained and distant relationships (Kildare & Middlemiss, 2017). In addition, prior research has linked this parent–child conflict to a higher risk of problematic internet use among teenagers (J. Zhou et al., 2022). From the perspective of compensatory internet use theory (Kardefelt-Winther, 2014), adolescents naturally seek support and warmth from their parents at home. When these emotional needs are not met offline, for instance due to frequent conflict, they may turn to cyberspace instead. Specifically, adolescents are more likely to rely on smartphones for comfort in situations where family relationships are strained and parental warmth is scarce.

In addition, Domoff et al.’s (2020) framework on problematic media use suggests that conflict between parents and children could function as a bridge linking technoference and adolescents’ smartphone addiction. According to this theory, children’s problematic smartphone use results from both personal traits and environmental factors. Specifically, parental technology use during family communication can blur family boundaries regarding smartphone rules and reduce the quality of communication between parents and children (Braune-Krickau et al., 2021; Komanchuk et al., 2023), thereby leading to more parent–child conflict. Consequently, children may turn to increased smartphone use to fill unmet psychological needs in the face of increased parent–child conflict due to perceived parental technoference. Thus, this study hypothesized that parent–child conflict operates as an intermediary variable in the associations linking technoference with adolescent smartphone addiction.

### 1.3. Mindfulness as a Moderator

As Kabat-Zinn (2003) describes, mindfulness is essentially the conscious awareness of the present moment, observing all experiences without judgment. Engaging with all experiences—positive, negative, or neutral—in this non-judgmental way can reduce distress and enhance well-being (Germer, 2004). A growing body of literature indicates that mindfulness tends to show positive correlations with academic achievement (Bordbar et al., 2024), self-esteem (K. Wang & Kong, 2020), psychological well-being (Singh et al., 2016), and life satisfaction (Yüksel Doğan & Metin, 2023) and that it is negatively associated with depression (Sharma & Kumra, 2022), anxiety and stress (Bajaj et al., 2016), and difficulties in social functioning (W. Xu et al., 2018). Importantly, research has indicated that mindfulness may serve as a potential buffering factor for addictive behaviors. Individuals reporting higher mindfulness levels tend to demonstrate a diminished engagement in problematic internet behaviors, or smartphone addiction (Calvete et al., 2017; X. Li & Hao, 2019).

The stress-buffering hypothesis suggests that positive personal qualities (e.g., mindfulness) can alleviate the negative impact of difficult life events (Wood et al., 2010). Essentially, mindfulness enables individuals to navigate adversity more skillfully by helping them regulate their emotions, thought patterns, and actions, thereby serving as a psychological shield against stress (McKeen et al., 2023). Liang et al. (2023) reported that adolescents with higher levels of mindfulness were less to become dependent on their smartphones, even when subjected to father phubbing (i.e., their fathers being distracted by their smartphones), suggesting that mindfulness may buffer the relationship between parental distraction and excessive smartphone engagement. Similarly, X. Li and Hao (2019) observed that adolescents with high alexithymia exhibited heavier reliance on phones, but only if they lacked mindfulness, again pointing to a possible moderating role of mindfulness. Guided by these insights, the present study hypothesized that mindfulness moderates the relationships between perceived parental technoference and attentional shifting, parent–child conflict, and adolescent smartphone addiction.

### 1.4. The Present Study

Although existing literature has consistently identified links perceived parental technoference and adolescent smartphone addiction, several gaps remain. First, while the framework of childhood problematic media use proposed by Domoff et al. (2020) highlights the importance of the combined influences of personal attributes and contextual factors, empirical support for these models in the context of smartphone addiction is still limited. Previous studies have typically examined either individual- or family-level mechanisms in isolation, with few attempts to integrate both perspectives into a comprehensive model. Moreover, research has primarily focused on potential mediators such as social anxiety or self-control, with relatively little attention given to cognitive variables such as attentional shifting.

**Figure 1:**
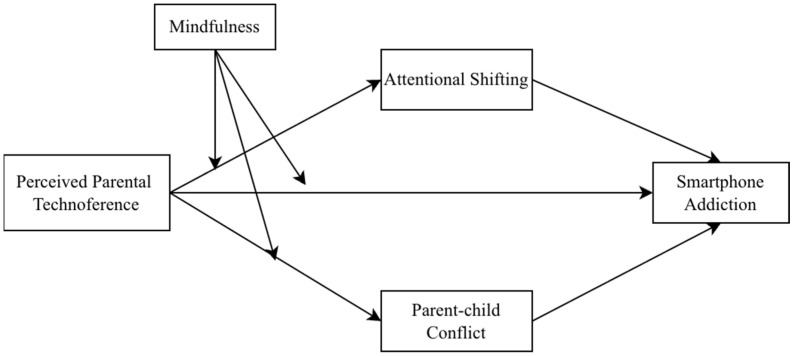
Conceptual framework.

Accordingly, the objective of the present study is to test an integrated moderated mediation model linking adolescents’ perceived parental technoference to smartphone addiction by examining (a) the mediating roles of attentional shifting and parent–child conflict, and (b) whether mindfulness moderates these associations. Figure 1 illustrates this study’s conceptual framework. The following research hypotheses are proposed:

**H1.**  *Perceived parental technoference is associated with adolescents’ smartphone addiction*.

**H2.**  *Perceived parental technoference is indirectly associated with adolescents’ smartphone addiction through attentional shifting*.

**H3.**  *Perceived parental technoference is indirectly associated with adolescents’ smartphone addiction through parent–child conflict*.

**H4.**  *Mindfulness moderates the associations between perceived parental technoference and attentional shifting, parent–child conflict, and smartphone addiction*.

## 2. Methods

### 2.1. Participants and Procedure

This study employed a cross-sectional, quantitative correlational design and collected data using self-report questionnaires. Multi-stage cluster random sampling was adopted because the target population is naturally clustered within schools and classes in real-world settings. In addition, this approach retains the probabilistic features of random selection across multiple stages (e.g., district–school–class). While balancing feasibility and cost, it helps improve sample representativeness and increase sample diversity and heterogeneity. Specifically, participants were recruited from six schools in Handan City, Hebei Province, China. With the assistance of the local education bureau, two districts were first randomly selected from Handan City. Then, within each district, two primary schools, two junior high schools, and two senior high schools were randomly selected. Given that students in Grades 1–3 may have limited ability to complete questionnaires independently, only students in Grades 4–6 were invited to participate. Within each selected school, two classes were randomly selected from each grade, and all students in the selected classes were invited to complete the survey. Prior to implementation, the research team coordinated with schools in advance and administered the questionnaires during a self-study period. During data collection, trained research assistants were present in the classroom to provide necessary instructions and answer questions. Before data collection began, informed consent was obtained from both students and their guardians, and the study was approved by the university’s ethics review board.

Compared with simpler models, complex models with more parameters typically require larger sample sizes to achieve adequate statistical power and stable parameter estimates (Kline, 2023). Simulation evidence from Fritz and MacKinnon (2007) shows that mediation analyses often require large samples, particularly when the path effects are small. For example, a bias-corrected bootstrap test of the indirect effect requires approximately 462 cases, and some alternative testing approaches may require up to 667 cases. Given that the moderated mediation model in the present study is more complex than a simple mediation model, a larger sample is therefore necessary to ensure adequate power and robust estimation. In total, 1084 valid questionnaires were collected. The final sample included 47% boys and 53% girls, with a mean age of approximately 14 years (M = 13.980, SD = 2.099). In terms of school level, 24.8% of participants were from primary schools, 29.5% from middle schools, and 45.7% from high schools.

### 2.2. Measurements

#### 2.2.1. Perceived Parental Technoference

To measure perceived parental technoference, we used the Chinese version of the Technology Interference with Life Examples Scale (McDaniel & Coyne, 2016; Zhong et al., 2023). Participants were asked to assess their parents’ behaviors over the past 12 months across five items, such as “During a typical mealtime that my parents and I spend together, my parents pull out and check their phones or mobile devices”. Respondents rated each statement on a 5-point Likert scale ranging from “never” (1) to “always” (5). An average score was calculated, where higher scores suggesting a greater degree of technoference from parents. In the current study, the internal consistency of the scale was acceptable, with a Cronbach’s alpha of 0.754 and a McDonald’s omega of 0.755.

#### 2.2.2. Attentional Shifting

To assess adolescents’ ability to shift their attention, the attentional shifting subscale of the Attentional Control Scale (Derryberry & Reed, 2002) was used. Participants were asked to reflect on their experiences over the past 6 months across 11 items, such as “It’s very hard for me to concentrate on a difficult task when there are noises around.” Each item was rated on a 5-point Likert scale (1 = never, 5 = always). Responses were reverse-scored, so that higher mean scores represented stronger attentional shifting ability. One example item is “It’s very hard for me to concentrate on a difficult task when there are noises around”. Previous research has indicated that this measure shows acceptable reliability, and it has been validated in the Chinese context (W. H. Yang & He, 2019). In the current sample, this scale showed good reliability, with a Cronbach’s alpha of 0.870 and a McDonald’s omega of 0.868.

#### 2.2.3. Parent–Child Conflict

Parent–child conflict was measured using the Conflict Behavior subscale (Kleinschlömer & Krapf, 2023), an adapted version of an earlier instrument developed to measure children’s interpersonal relationships (Furman & Buhrmester, 1985). This measure is composed of two items: “how often your parents and you are angry with each other” and “how often your parents and you disagree and quarrel”. Participants were instructed to select the responses most suitable for their situation over the past 6 months on a 5-point Likert scale ranging from “never” (1) to “always” (5). Higher average ratings on the scale indicate more frequent conflict between adolescents and their parents. In this study, the measure demonstrated acceptable internal consistency, with a Cronbach’s alpha of 0.825.

#### 2.2.4. Smartphone Addiction

To evaluate smartphone addiction, we employed the validated Chinese adaptation of the smartphone addiction scale (Kwon et al., 2013; M. Zhou et al., 2023). Participants were asked to evaluate their smartphone use over the past month across 10 items, including statements like “Won’t be able to stand not having a smartphone”. Respondents rate each statement on a 6-point Likert scale, from 1 (strongly disagree) to 6 (strongly agree). A composite score was calculated by averaging item responses, with higher scores indicating more severe smartphone dependence. In the current study, the Cronbach’s alpha was 0.836 and the McDonald’s omega was 0.838, reflecting strong internal consistency.

#### 2.2.5. Mindfulness

Adolescents’ mindfulness levels were measured using the Chinese version of the mindfulness subscale (J. Chen et al., 2011; Neff, 2003). It includes four items, such as “When something upsets me, I try to keep my emotions in balance”. Responses were recorded on a 5-point Likert scale ranging from 1 (never) to 5 (always). Overall mindfulness was assessed by averaging the scores across all items, with higher values reflecting a greater degree of mindfulness. In this sample, the scale demonstrated high internal consistency, with both Cronbach’s alpha and McDonald’s omega equal to 0.849.

#### 2.2.6. Covariates

Following prior research on perceived parental technoference and smartphone addiction (X. Chen et al., 2020; Q. Liu et al., 2020), we incorporated gender (1 = male, 0 = female), age, and subjective economic status (1 = very difficult, 5 = well-off) as covariates in our analytical models. Specifically, empirical evidence suggests that these demographic characteristics are closely associated with both the predictor (i.e., perceived parental technoference) and the outcome (i.e., smartphone addiction), making them important potential confounders.

Regarding gender, girls are typically more sensitive to relational and emotional cues and place greater value on parent–child interaction, which is associated with higher perceived parental technoference (Shao et al., 2024). Evidence on gender differences in smartphone addiction is mixed (Fischer-Grote et al., 2019). Recent studies have begun to focus on the purposes of smartphone use in smartphone addiction, finding that girls are more inclined to use smartphones for social media, chatting, and watching short videos, whereas boys tend to prefer gaming or watching videos (Peng et al., 2025).

Age is also associated with these variables. As adolescents grow older, their expectations for emotional support and respect in parent–child interactions tend to increase; correspondingly, older adolescents may be more likely to perceive parents’ phone use during interactions as technoference (Knitter & Zemp, 2020). In addition, age is associated with greater autonomy and stronger peer and social-media needs, which are related to greater reliance on smartphones and a higher risk of addictive use (Azizi et al., 2024).

Findings regarding subjective economic status are mixed. Higher status is associated with parents’ greater digital literacy and stronger device-management capacity, which correspond to a lower risk of parental technoference (Leon & Cilich, 2025). Conversely, lower status is associated with parents’ greater reliance on technology for information, support, or substitute caregiving, which is linked to a higher risk of parental technoference (Tarigan & Setiasih, 2025). For smartphone addiction, some studies report higher levels among higher-income adolescents because they face fewer resource constraints (Long et al., 2016), whereas others report higher vulnerability among lower-status adolescents, which may be associated with stress, relative deprivation, and parents’ limited time for after-school involvement (Lin & Liu, 2020; Yun, 2021).

Accordingly, to reduce potential confounding and more clearly examine the association between perceived parental technoference and adolescent smartphone addiction, we included gender, age, and subjective economic status as control variables in all subsequent analyses.

### 2.3. Data Analysis

Harman’s one-factor test was employed to examine whether common method bias existed. Following standard guidelines, common method variance would not substantially compromise the validity of our findings if the first common factor accounted for under 50% of the total variance (Kock et al., 2021). Descriptive statistics and bivariate correlations were conducted using SPSS 31.0. To test for mediating and moderating effects, we used SPSS’s macro PROCESS 5.0 (Hayes, 2017). Specifically, Model 4 was used to assess whether attentional shifting and parent–child conflict act as intermediary variables linking perceived parental technoference to smartphone addiction. We generated a bootstrap resampling procedure with 5000 iterations to generate a 95% confidence interval (CI) for the mediation effect, and statistical significance was confirmed if zero fell outside these intervals (Hayes, 2017). Moreover, we used Model 8 to explore whether mindfulness moderated the relationship between technoference and smartphone addiction. To gain a clearer picture of this moderating effect, we performed a simple slope analysis and visualized the findings using plots. Additionally, gender, age, and subjective economic status were included as covariates in the analysis to control for potential confounding variables.

## 3. Results

### 3.1. Common Method Bias and Preliminary Analysis

Harman’s single-factor test revealed eight factors with eigenvalues exceeding 1. The first factor explained 24.16% of the total variance—well under the 50% benchmark that typically signals common method bias.

Descriptive statistics and Pearson correlations among variables are shown in Table 1. Perceived parental technoference demonstrated a negative association with attentional shifting (r = −0.295, *p* < 0.001) but was positively linked to both parent–child conflict (r = 0.329, *p* < 0.001) and smartphone addiction (r = 0.286, *p* < 0.001). No significant correlation was found between perceived parental technoference and mindfulness (r = 0.009, *p* > 0.05). Meanwhile, attentional shifting showed inverse relationships with parent–child conflict (r = −0.377, *p* < 0.001) and smartphone addiction (r = −0.488, *p* < 0.001), while positively correlating with mindfulness (r = 0.103, *p* < 0.001). Parent–child conflict was positively tied to smartphone addiction (r = 0.311, *p* < 0.001) yet inversely related to mindfulness (r = −0.064, *p* < 0.05). Smartphone addiction exhibited a modest negative correlation with mindfulness (r = −0.118, *p* < 0.001).

### 3.2. Mediating Effect Analyses

The results of the mediation analysis, as shown in Table 2, suggest that perceived parental technoference had a significant positive association with smartphone addiction (B = 0.137, *p* < 0.001). Moreover, perceived parental technoference was found to be negatively linked to adolescents’ attentional shifting (B = −0.196, *p* < 0.001), which in turn was correlated with higher levels of smartphone addiction (B = −0.633, *p* < 0.001). Furthermore, as shown in Table 3, bootstrapping procedures confirmed that attentional shifting significantly mediated this association (B = 0.124, 95% CI [0.093, 0.158]). Similarly, perceived parental technoference showed a positive relationship with parent–child conflict (B = 0.392, *p* < 0.001), which was also positively associated with smartphone addiction (B = 0.091, *p* < 0.001). The mediating effect of parent–child conflict was statistically significant (B = 0.036, 95% CI [0.014, 0.058]; see Table 3), highlighting its potential role in the link between perceived parental technoference and smartphone addiction.

### 3.3. Moderated Mediation Analyses

**Figure 2:**
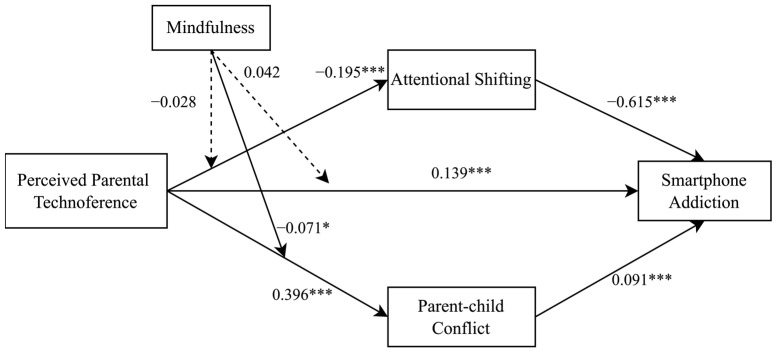
Standardized coefficients for the moderated mediation model. Solid arrows represent significant paths, and dashed arrows represent non-significant paths; * *p* < 0.05, *** *p* < 0.001.

**Figure 3:**
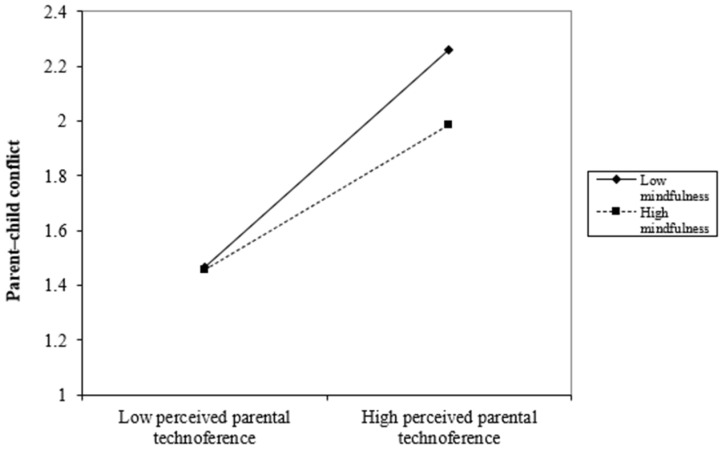
Moderating role of mindfulness between perceived parental technoference and parent–child conflict.

The results of the moderated mediation analyses are displayed in Table 4 and Figure 2. Our findings reveal a statistically significant interaction effect between perceived parental technoference and mindfulness in relation to parent–child conflict (B = −0.085, *p* < 0.05), indicating that mindfulness served as a buffer in this dynamic. However, no significant moderating effect emerged for either attentional shifting (B = −0.019, *p* > 0.05) or smartphone addiction (B = 0.046, *p* > 0.05), indicating that mindfulness did not significantly moderate these pathways. Furthermore, we plotted parent–child conflict prediction scores against perceived parental technoference at low and high levels of mindfulness (defined as one standard deviation below and above the mean, respectively; see Figure 3). The analysis revealed that adolescents with lower mindfulness levels (B_simple_ = 0.490, *p* < 0.001) experienced a stronger association between perceived parental technoference and parent–child conflict, compared to their counterparts with higher mindfulness (B_simple_ = 0.321, *p* < 0.001).

## 4. Discussion

Drawing upon the interactional theory of childhood problematic media use, the compensatory internet use theory, and the stress-buffering hypothesis, this research explores how perceived parental technoference and smartphone addiction are linked. The findings reveal the importance of attentional shifting, parent–child conflict, and mindfulness in this relationship, providing a comprehensive framework for understanding adolescent smartphone addiction.

### 4.1. Perceived Parental Technoference and Smartphone Addiction

The results indicate that perceived parental technoference is closely linked to higher levels of smartphone addiction among adolescents. This is aligned with a previous study (Geng et al., 2021), which emphasizes the critical role of parental technology use in adolescents’ tendencies toward excessive smartphone use. These findings also consistent with Bandura’s (1971) social learning theory, which holds that individuals develop behaviors by observing and emulating key figures in their lives, particularly their parents. When parents frequently use smartphones in their daily lives, this parental dependence on technology provides a model for adolescents’ addiction to smartphones (Andreassen, 2015). Over time, such exposure could be associated with increased identification with and imitation of parental smartphone use, potentially contributing to adolescents’ problematic engagement with smartphones.

### 4.2. Mediating Effect of Attentional Shifting 

The results of this study indicate that attentional shifting could potentially function as a potential mediator linking parental technoreference with adolescent smartphone addiction. Specifically, adolescents who perceived their parents as being frequently distracted by technology tended to demonstrate lower attentional shifting abilities in adolescents, which, in turn, were related to greater tendencies toward compulsive smartphone use. These findings align with Domoff et al.’s (2020) framework on childhood media overuse, which emphasizes the combined influence of individual characteristics (e.g., emotion regulation) and contextual interactions (e.g., parental behaviors) on problematic media use. Parental technology use may be associated with adolescents’ emotion regulation capacities, such as attentional shifting, which could help explain their greater susceptibility to problematic smartphone use. Similarly, the present findings corroborate previous evidence indicating that perceived parental technoference may be linked to reduced emotional responsiveness and behavioral consistency within the family environment (Braune-Krickau et al., 2021), and that such relational patterns may be associated with impairments in adolescents’ attentional functioning (McDaniel, 2019) and emotion regulation (Xie et al., 2020). Adolescents with lower attentional shifting abilities may be more prone to engage with highly stimulating or distracting digital content, such as smartphone, which is linked to an elevated likelihood of compulsive usage (Billieux et al., 2015; Wilmer et al., 2017).

### 4.3. Mediating Effect of Parent–Child Conflict

The current results align with prior literature (S. Liu et al., 2024), indicating that conflicts between parents and children might serve as a potential intermediary factor in the link between technoference and adolescents’ problematic smartphone use. Adolescents who observe their parents heavily engaged with digital devices during family time are more likely to report elevated levels of conflict with their parents. This heightened conflict, in turn, is associated with greater tendencies toward problematic smartphone use among adolescents. This finding aligns with the individual × context interaction framework (Lerner et al., 2006), which posits that adolescents’ smartphone overuse is influenced not only by individual factors but also by family dynamics. Perceived parental technoference, as a form of negative family environment (C. Xu & Xie, 2023), disrupts face-to-face interactions (Xie et al., 2019) and undermines the family support system, thereby intensifying parent–child conflict (Shen et al., 2022). In this context, children may make heavy use of smartphones to fill the unmet needs caused by increased conflict with their parents. Similarly, according to the compensatory internet use theory (Kardefelt-Winther, 2014), perceived parental technoference and frequent parent–child conflicts diminish adolescents’ perceived familial warmth and emotional support. In response to these unmet psychological needs, adolescents are more likely to turn to smartphones as alternative sources of emotional gratification (Park et al., 2008). Over time, this compensatory reliance on digital devices can lead to maladaptive smartphone use habits (Stockdale et al., 2018).

### 4.4. Moderating Effect of Mindfulness 

Our study also suggests that mindfulness serves as a buffering mechanism that mitigates the harmful consequence of parental technoreference on parent–child conflict. Notably, in our sample, adolescents with weaker mindfulness skills experienced more pronounced increases in parent–child conflict due to perceived parental technoference compared to their more mindful peers. The results are congruent with the stress-buffering hypothesis (Rueger et al., 2016), which holds that psychological resources (e.g., mindfulness) are pivotal in mitigating the negative effects of stressors on individuals’ developmental outcomes. Mindfulness, often described as a mental skill that promotes healthy stress management (Gilbert et al., 2017; Keng et al., 2011), equips adolescents to handle perceived parental technoference with greater composure. When experiencing perceived parental technoference, adolescents with stronger mindfulness capacities tend to employ adaptive strategies like calm communication or delayed discussion, rather than impulsive reactions like yelling (X. Chen & Deng, 2022), thereby reducing parent–child conflict.

However, mindfulness did not significantly attenuate the impact of perceived parental technoference on attentional shifting in our study. One possible explanation is that attentional shifting, as a core component of executive functioning, primarily relies on neurocognitive mechanisms at the physiological level such as the frontoparietal network (Petersen & Posner, 2012; Tamber-Rosenau et al., 2011), rather than on subjective self-regulatory capacities such as mindfulness. Given this biological foundation, mindfulness appears to have limited power to counteract how perceived parental technoference disrupts adolescents’ attentional shifting. Consequently, when perceived parental technoference diminishes adolescents’ ability to shift attention, adolescents may find it difficult to compensate for this deficit through mindfulness practices.

The study also found that mindfulness did not significantly moderate the direct association between perceived parental technoference and adolescent smartphone dependency. A plausible interpretation for this finding is that mindfulness primarily operates by enhancing emotional and mood regulation (Cheng et al., 2020; D. Li et al., 2023), thereby indirectly reducing susceptibility to problematic smartphone use. This implies that the protective effects of mindfulness may not be directly observable in the pathway from perceived parental technoference to smartphone addiction. Rather, mindfulness appears to exert a stronger influence on the indirect pathway—for example, by alleviating the adverse effects of parental technoreference on increasing parent–child conflict, which in turn contributes to addiction. In this sense, the moderating role of mindfulness in the direct association may be obscured, as it functions primarily by weakening the mediating mechanisms (e.g., parent–child conflict) that underlie connect perceived parental technoference to smartphone addiction.

## 5. Limitations

This research comes with several noteworthy limitations. First, the cross-sectional design limits the ability to establish casual relationships. Longitudinal studies are recommended to better elucidate how these variables interact over time. Second, relying solely on adolescents’ self-reported data may introduce response bias and limit internal validity. Future studies should incorporate perspectives from parents, teachers, and peers to provide a more complete picture. Third, the sample was only from one city in China; thus, the findings may not be generalizable to other geographic or cultural populations. Moreover, prior research has demonstrated that maternal and paternal technoference exert differential effects on adolescents’ externalizing and internalizing behaviors (McDaniel & Radesky, 2018b). Thus, it is critical that future studies independently explore the respective impacts of father and mother technoference on adolescents’ smartphone addiction. Additionally, other parenting-related variables (e.g., parental mediation) may function as potential confounding variables in the relationship between technoference and smartphone addiction. Unfortunately, these variables have not yet been fully explored or adequately controlled. Future research should incorporate a broader spectrum of parenting factors, which may offer novel insights into the mechanisms underlying adolescent smartphone addiction.

## 6. Implications

This study offers several important implications. Theoretically, it adds to existing scholarship by proposing an integrated model of adolescent smartphone addiction, outlining how perceived parental technoference relate to addiction-related outcomes through both individual cognitive factors (i.e., attentional shifting) and family dynamic factors (i.e., parent–child conflict). The model provides insight into the underlying processes connecting perceived parental technoference with adolescent smartphone use.

On a practical level, the findings the results draw attention to the detrimental consequences of perceived parental technoference and underscore the importance of developing parental education programs by social workers to raise awareness of digital device overuse in parent-adolescent interactions. Given the pervasive role of digital devices in daily life, complete avoidance is unrealistic (Dixon et al., 2023). Therefore, community social workers can support families in developing mutually agreed upon rules to manage device use (Kutrovátz, 2022), lowering adolescents’ susceptibility to problematic smartphone dependence. Second, the role of attentional shifting as a potential mediating mechanism suggests that social work interventions should incorporate strategies to strengthen adolescents’ attention control and cognitive self-regulation. School social workers and mental health practitioners may integrate attention-enhancement techniques into prevention programs targeting adolescents’ addiction. Third, the finding that parent–child conflict serves as a bridge linking technoference and addiction emphasizes the importance of interventions that target family interaction patterns. Programs such as Parent–Child Interaction Therapy (Lee & Lee, 2023) can be adapted by social workers to include “tech-aware parenting” modules—such as maintaining emotional presence during device use and establishing technology-free routines (McDaniel & Radesky, 2018a)—to foster healthier and more communicative family environments. Finally, the potential of mindfulness as a protective factor suggests that social work practitioners and school-based counselors could develop or implement evidence-based mindfulness programs, such as Learning to BREATHE (Bluth et al., 2015). This program has been shown to effectively enhance emotional awareness and alleviate stress through the practice of present-focused, non-judgmental attention. By cultivating these core mindfulness skills, such interventions can help adolescents improve emotion regulation and reduce relational strain associated with digital distractions in family settings.

## 7. Conclusions

This study examined the associations among perceived parental technoference, attentional shifting, parent–child conflict, smartphone addiction, and mindfulness. The findings revealed that perceived parental technoference was negatively associated with adolescents’ smartphone addiction, and this relationship was mediated by attentional shifting and parent–child conflict. Specifically, perceived parental technoference may undermine adolescents’ attentional shifting and intensify parent–child conflict, which in turn were associated with higher levels of adolescents’ smartphone addiction. In addition, mindfulness served as a protective factor by moderating the link between perceived parental technoference and parent–child conflict. Adolescents with lower mindfulness experienced stronger increases in parent–child conflict when perceiving higher levels of parental technoference. Taken together, these results underscore the importance of integrating individual cognitive processes and family dynamics when explaining adolescent smartphone addiction and highlight mindfulness as a potential target for interventions aimed at reducing family conflict and problematic smartphone use in the context of parental technoference.

## Figures and Tables

**Table 1:** Descriptive statistics and correlations for variables. Note: PPT = Perceived parental technoference, AS = Attentional shifting, PC = Parent–child conflict, SA = Smartphone addiction, MF = Mindfulness; * *p* < 0.05, ** *p* < 0.01. *** *p* < 0.001.

	M	SD	1	2	3	4	5
1. PPT	2.289	0.834	1				
2. AS	2.935	0.564	−0.295 ***	1			
3. PC	2.398	0.987	0.329 ***	−0.377 ***	1		
4. SA	3.011	0.900	0.286 ***	−0.488 ***	0.311 ***	1	
5. MF	3.114	0.941	0.009	0.103 ***	−0.064 *	−0.118 **	1

**Table 2:** Results of the mediation model. Note: SES = Subjective economic status, PPT = Perceived parental technoference; * *p* < 0.05, ** *p* < 0.01, *** *p* < 0.001.

Predictors	Attentional Shifting			Parent–Child Conflict			Smartphone Addiction		
	B	β	SE	B	β	SE	B	β	SE
Age	−0.019 *	−0.07	0.008	0.054 ***	0.116	0.013	0.055 ***	0.127	0.011
Gender	0.051	0.045	0.033	−0.225 ***	−0.114	0.056	−0.045	−0.025	0.047
SES	0.066	0.057	0.034	−0.036	−0.018	0.059	−0.128 **	−0.069	0.049
PPT	−0.196 ***	−0.29	0.020	0.392 ***	0.332	0.034	0.137 ***	0.127	0.030
Attentional shifting							−0.633 ***	−0.396	0.045
Parent–child conflict							0.091 ***	0.099	0.026
R^2^	0.097			0.133			0.293		
F	28.934 ***			41.431 ***			75.534 ***		

**Table 3:** Bootstrap analysis of direct and indirect effects. Note: CI = confidence intervals.

Effect	B	β	SE	t	*p*	95% CI	
						Lower	Upper
Direct effect: Perceived parental technoference → Smartphone addiction	0.137	0.127	0.030	4.555	<0.001	0.078	0.196
Indirect effect: Perceived parental technoference → Attentional shifting → Smartphone addiction	0.124	0.115	0.017	−	−	0.093	0.158
Indirect effect: Perceived parental technoference → Parent–child conflict → Smartphone addiction	0.036	0.033	0.011	−	−	0.014	0.058
Total effect	0.297	0.275	0.031	9.571	<0.001	0.236	0.358

**Table 4:** Results of the moderated mediation model. Note: SES = Subjective economic status, PPT = Perceived parental technoference, MF = Mindfulness, PPT × MF = Interaction term; * *p* < 0.05, ** *p* < 0.01, *** *p* < 0.001.

Predictors	Attentional Shifting			Parent–Child Conflict			Smartphone Addiction		
	B	β	SE	B	β	SE	B	β	SE
Age	−0.023 **	−0.085	0.008	0.057 ***	0.120	0.014	0.060 ***	0.141	0.011
Gender	0.038	0.034	0.033	−0.199 ***	−0.101	0.057	−0.033	−0.018	0.047
SES	0.057	0.049	0.034	−0.033	−0.016	0.059	−0.117 *	−0.062	0.048
PPT	−0.195 ***	−0.289	0.020	0.396 ***	0.335	0.034	0.139 ***	0.129	0.030
Attentional shifting							−0.615 ***	−0.385	0.045
Parent–child conflict							0.091 ***	0.100	0.026
Mindfulness	0.065 ***	0.109	0.018	−0.076 *	−0.072	0.030	−0.080 **	−0.084	0.025
PPT × MF	−0.019	−0.028	0.020	−0.085 *	−0.071	0.034	0.046	0.042	0.028
R^2^	0.109			0.143			0.302		
F	22.049 ***			29.869 ***			58.192 ***		

## Data Availability

Data will be made available on request.
